# Microalgae growth in ultra-thin steady-state continuous photobioreactors: assessing self-shading effects

**DOI:** 10.3389/fbioe.2022.977429

**Published:** 2022-08-11

**Authors:** Alberto Saccardo, Fabrizio Bezzo, Eleonora Sforza

**Affiliations:** ^1^ CAPE-Lab (Computer-Aided Process Engineering Laboratory), Department of Industrial Engineering, University of Padova, Padova, Italy; ^2^ Department of Industrial Engineering, University of Padova, Padova, Italy

**Keywords:** *Scenedesmus obliquus*, *Tetradesmus obliquus*, mixing cycles, light path, growth modeling, continuous photobioreactor

## Abstract

To disclose the net effect of light on microalgal growth in photobioreactors, self-shading and mixing-induced light–dark cycles must be minimized and discerned from the transient phenomena of acclimation. In this work, we performed experiments of continuous microalgal cultivation in small-scale photobioreactors with different thicknesses (from 2 to 35 mm): working at a steady state allowed us to describe the effect of light after acclimation, while the geometry of the reactor was adjusted to find the threshold light path that can discriminate different phenomena. Experiments showed an increased inhibition under smaller culture light paths, suggesting a strong shading effect at thicknesses higher than 8 mm where mixing-induced light–dark cycles may occur. A Haldane-like model was applied and kinetic parameters retrieved, showing possible issues in the scalability of experimental results at different light paths if mixing-induced light–dark cycles are not considered. To further highlight the influence of mixing cycles, we proposed an analogy between small-scale operations with continuous light and PBR operations with pulsed light, with the computation of characteristic parameters from pulsed-light microalgae growth mathematical modeling.

## 1 Introduction

Among all the factors that affect microalgae productivity and growth, light covers a major role since it provides all the energy required for metabolism. The effect of light on microalgal photosynthesis has been the subject of a number of studies in the past that identify three different light-dependent regions for microalgal growth: photolimitation, photosaturation, and photoinhibition.

The experimental observation of these three regions in photobioreactors is influenced by two adverse phenomena that hinder the true photosynthetic response to light: self-shading and mixing-induced light–dark cycles (Barbosa et al., 2003; Graham et al., 2015). Self-shading is light attenuation by microalgae cell absorption that reduces light availability in the inner parts of the photobioreactors (Flynn, 2021). Moreover, microalgae are exposed to light–dark cycles because of the light gradient induced by the self-shading and the turbulent mixing in the reactor; due to these conditions, cells receive light intermittently (Barbosa et al., 2003; Zarmi et al., 2013). A strategy to minimize these adverse phenomena is to decrease the culture light path (Castaldello et al., 2019).

Decreasing the scale of photobioreactors (to microphotobioreactors) can lead to multiple advantages, such as faster experiments in large numbers of replicas with the possibility to precisely control growth conditions (Perin et al., 2016; Kim et al., 2018). Microphotobioreactors can reach scale volumes down to the pico-liter (Kim et al., 2018) and have been used to cultivate microalgae in droplet-based flow systems (Saad et al., 2019a; 2019b), single-cell layer microfluidic chips (Luke et al., 2016; Westerwalbesloh et al., 2019), or fed-batch microwell systems (Perin et al., 2016; Castaldello et al., 2019). Recently, microphotobioreactors have been especially used to investigate light effects on microalgal growth, that is, applications include microalgal growth under irradiance and nitrate stress conditions (Saad et al., 2019a; 2019b) and the evaluation of light effects on microalgae culture under non-limiting CO_2_ conditions (Castaldello et al., 2019). A limitation of the aforementioned works is that due to the micro-scale, continuous operations are not possible. This could be achieved by operating on a small scale (instead of a micro-scale) that represents an intermediate between microphotobioreactors and traditional “high-scale” systems. Several attempts have been made in previous literature, such as using a flat plate photobioreactor of 2 cm thickness by Busnel et al. (2021) or Pfaffinger et al. (2019). Other studies researched steady-state reactors of 1.2–1.5 cm deep (Sforza et al., 2014, 2015a, 2015b, 2018; Barbera et al., 2017), finding remarkably high concentrations, confirming that self-shading is limited. However, it is not clear if a light path of 1.5–2 cm is actually able to avoid the self-shading effect.

The objective of this work was to capture the net effect of light on microalgal growth in photobioreactors by minimizing phenomena of self-shading and mixing-induced light–dark cycles in acclimated cultures. For this purpose, we designed small-scale continuous closed photobioreactors with different reactor thicknesses of 2, 5, and 8 mm, where *Tetradesmus obliquus* was cultivated at a steady state. The employment of such a small scale (2–8 mm thickness of the light path) in a continuous mode represents a novelty of this work; to our knowledge, few attempts of microalgae cultivations in closed photobioreactors with these light paths are reported in the literature and none in the continuous mode. Thin-layer cascade (TLC) photobioreactors can exhibit light paths of a few millimeters, with the possibility of semi-continuous or continuous operation (Grivalský et al., 2019). However, TLCs are generally operated *via* flow recirculation usually comprising a retention chamber (Grivalský et al., 2019). This operation mode determines that microalgae are not continuously exposed to light and make TLCs significantly different from continuous ultrathin flat-plate photobioreactors.

We compared our experimental data at 2–8 mm with those at 15 and 35 mm for the same species and similar cultivation systems (Sforza et al., 2015b; Barbera et al., 2017; Borella et al., 2021) to highlight the effect of self-shading and mixing-induced light cycles. A modeling approach was then used on all the experimental data to better assess the impact of self-shading with reference to the available light path.

## 2 Materials and methods

### 2.1 Microalga and cultivation systems

*Tetradesmus obliquus* 276–7 was cultivated in a sterile BG11 medium (Stanier et al*.*, 1979) (modified to have a double concentration of all the nutrients in order to avoid limitation and ensure that light was the only limiting factor), buffered with 10 mM HEPES pH 8 to keep pH of the culture between 7 and 8 and minimize the acidification provided by CO_2_. The exact composition of the growth medium is reported in Supplementary Material. We provided CO_2_ in excess in a 5% volumetric air flowrate from the bottom of the reactors. The reactors were kept in a refrigerated incubator at a constant temperature of 24 °C. An LED lamp (Light Source SL3500, Photon System Instruments, Czech Republic) was used to supply light, with tunable intensities measured by an HD of 2102.1 photoradiometer (Delta OHM, Italy).

All the photobioreactors were operated in the same CSTR (continuous stirred tank reactor) mode and have the same inlet–outlet structure, with an inlet liquid stream for nutrients, an inlet gaseous stream of CO_2_ in a 5% volumetric air flowrate, and a liquid outlet stream to maintain the same liquid level over time. However, the shape of the reactors can be different for building purposes. The reactors of 2 mm thickness and 1.5 ml volume were made of PDMS and have a V shape, with a bottom inlet for air, a lateral inlet for nutrients, and an overflow liquid exit. Mixing was ensured by bubbling. The reactors of 5 and 8 mm and 10 ml volumes were made of polycarbonate with a bottom U shape. Stainless steel needles were used for the gaseous and liquid inlets and sampling outlets. The liquid overflow outlet was connected to a peristaltic pump for better level control. Mixing was obtained through bubbling and with the aid of a small magnetic stirrer. For all reactors, a liquid inlet for the continuous supply of a fresh medium was ensured by a two-way PHD ULTRA syringe pump (Harvard apparatus, United States). Pictures of the reactors are shown in Supplementary Material. Cultivation systems in Barbera et al. (2017) (15 mm, 700 ml), Borella et al. (2021) (35 mm, 200 ml), and Sforza et al. (2015b) (15 mm, 250 ml) are similar to the aforementioned ones, with the exception that 1.5 and 3.5 cm light path reactors have a flat bottom rectangular shape.

### 2.2 Experiments and analytical procedures

With reactors from 2 to 8 mm, we performed experiments with different conditions of light and residence time as inputs and measured cell concentration as the output. The cell concentration was monitored daily through manual cell counting using a Bürker counting Chamber (Optik Labor, Germany). Data were recorded for at least 3 days since the culture reached a steady state. Since the small scale of the reactors did not allow performing reliable dry weight measurements, we converted measurements of cell concentration (cell/volume) into ponderal concentrations (mass of biomass/volume) by using a linear correlation with experimental cellular weight.

### 2.3 Mathematical model

To interpret our experimental data, we used a model by Bernard and Rémond (2012) presented in Barbera et al. (2020). Assuming a rectangular geometry, the light extinction profile is predicted by the Lambert–Beer law as:

$$I\left(z\right)=I_{0}\exp\left(-k_{a}X_{out}z\right)\text{, }$$ (1)

where $I_{0}$ ( ${\text{μmol m}}^{-2}{\text{ s}}^{-1})$ is the incident light intensity, $k_{a}$ ( ${\text{m}}^{2}{\text{g}}^{-1}$ ) is the Lambert–Beer light extinction coefficient, $X_{out}$ ( ${\text{g m}}^{-3}$ ) is the biomass concentration inside the reactor, and z ( m ) is the axial coordinate of the reactor depth. Since we model our PBR as a CSTR, $X_{out}$ is assumed homogeneous inside the reactor and thus constant along the culture depth. For the reactor geometries used, the mixing conditions were measured by tracer experiments, confirming that they can be reasonably assumed as CSTRs.

Since nutrients and CO_2_ are provided in excess, the biomass growth rate $r_{x}$ ( ${\text{g m}}^{-3}\;{\text{d}}^{-1}$ ) is a function of light only as an input.

$$r_{x}\left(z\right)=\mu_{max}\frac{I\left(z\right)}{I\left(z\right)+K_{I}{\left(\frac{I\left(z\right)}{I_{opt}}-1\right)}^{2}}X_{out}-k_{d}X_{out}\text{. }$$ (2)

In Eq. 2, $\mu_{max}$ ( ${\text{d}}^{-1}$ ) is the maximum specific growth rate; $K_{I}$ and $I_{opt}$ ( ${\text{μmol m}}^{-2}{\text{ s}}^{-1}$ ) are the half-saturation constant of the light response curve and the light intensity for maximal growth rate, respectively; and $k_{d}$ ( ${\text{d}}^{-1}$ ) is a specific decay rate that accounts for cell respiration and maintenance. The dependence of the biomass growth rate on light in Eq. 2 is a reparameterization of the Haldane cell growth model (Bernard and Rémond, 2012). Being dependent on the varying light intensity along the culture depth, biomass growth rate varies along the axial coordinate and can be integrated along the reactor depth to obtain an average biomass growth rate as

$${\bar{r}}_{x}=\frac{1}{W}\overset{W}{\underset{0}{\int }}r_{x}\left(z\right)dz\text{ ,}$$ (3)

where W ( m ) is the reactor thickness. The material balance on biomass can be written as

$$\frac{dX_{out}}{dt}=-\frac{1}{\tau }X_{out}+{\bar{r}}_{x}\text{, }$$ (4)

where τ ( d ) is the biomass residence time in the PBR. τ is defined as

$$\tau =\frac{V_{R}}{\dot{V}},$$ (5)

where $V_{R}$ ( ${\text{m}}^{3}$ ) is the volume of the reactor and $\dot{V}$ ( ${\text{m}}^{3}{\text{ d}}^{-1}$ ) is the liquid flowrate.

Inputs of the model of Eqs. 1–5 are τ and $I_{0}$ , the output is $X_{out}$ , and model parameters are $\mu_{max}$ , $K_{I}$ , $I_{opt}$ , $\;k_{d},\;k_{a}$ . Another known variable of the model is the reactor thickness W , which could also be treated as an input since it changes for different experiments.

With the model in Eqs. 1–5, we fitted experimental data presented in the following section. For parametric regression, we used a maximum likelihood algorithm on the software gPROMS (by Siemens Process Systems Engineering).

## 3 Results

### 3.1 Experimental results

**FIGURE 1:**
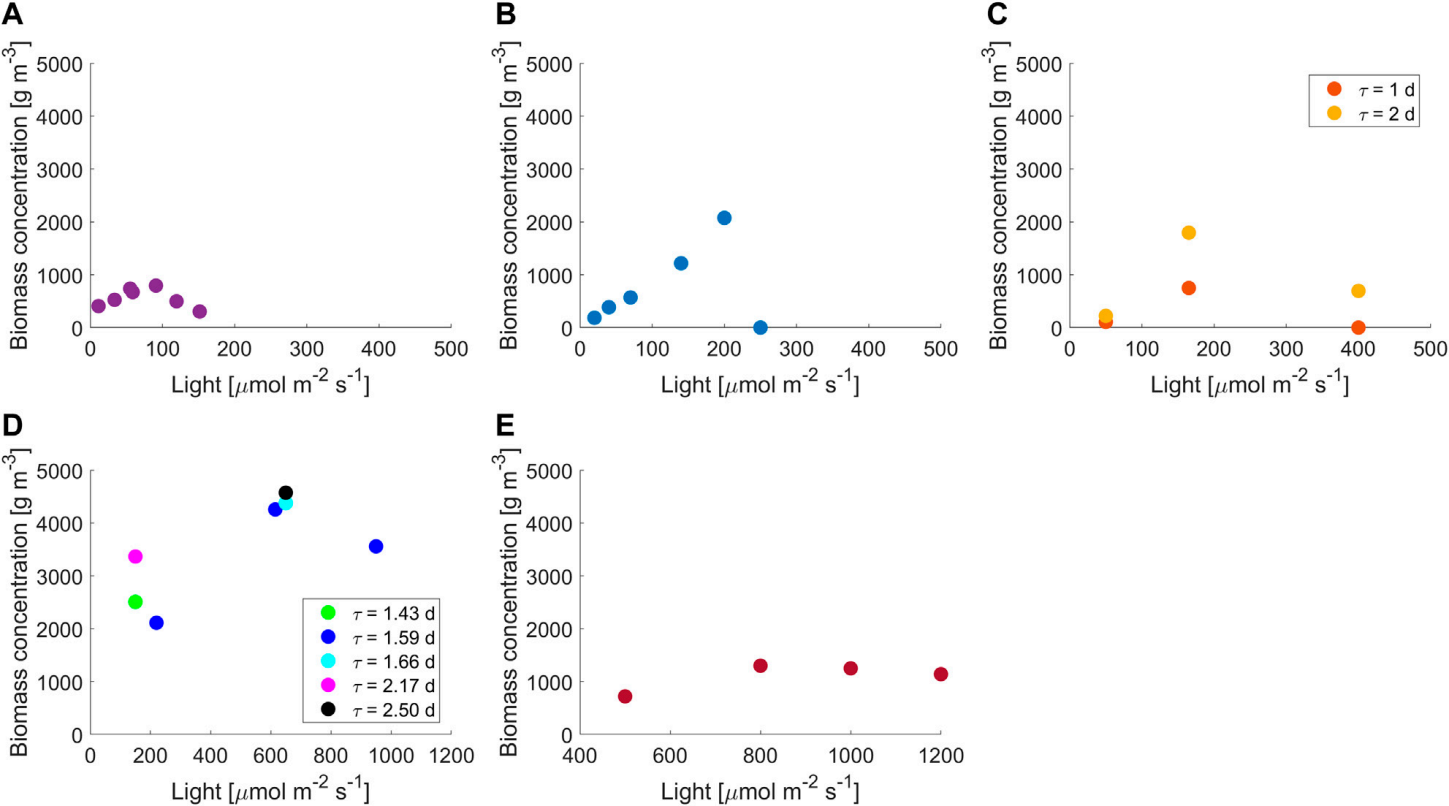
Experimental results at different light intensities and residence times for reactor thicknesses of **(A)** 2 mm, 1.5 days; **(B)** 5 mm, 1 day; **(C)** 8 mm, 1–2 days; **(D)** 15 mm, 1.43–2.5 days **(E)** 35 mm, 1.05 days. Data at 15 mm are from Barbera et al. (2017) and Sforza et al. (2015b); data at 35 mm are from Borella et al. (2021).

Biomass concentration was measured at a steady state in reactors with different light paths and as a function of light intensity and residence time, depending on the volume of the photobioreactor and the technological limits of the pumps used; experimental results are reported in Figure 1. Continuous small-scale photobioreactors opened new possibilities for the investigation of light-driven effects but require particular attention when operated. Due to the small volume, microalgae cultivation is highly sensitive to perturbations of the system that can eventually lead to the failure of the experiment. According to our experience, failures can occur either because of washout or because of the formation of irreversible biofouling. The arrangement of the reactor is critical: segregated zones should be avoided; materials must be biocompatible, with optimal optical properties and not sticky to minimize biofouling; and the mixing system (e.g., the magnetic stirrer or intensive bubbling) must not damage cells. Furthermore, monitoring operations must be performed carefully, and the sampling volumes should be carefully chosen in order to avoid introducing harmful perturbances (e.g., during the residence time) and exposing the culture to contaminations. What is mentioned earlier becomes more and more critical as the light path and volume of the reactor decrease. For instance, we were unable to reach steady-state conditions for light paths below 2 mm and reactor volumes below 1.5 ml.

The stability of the small-scale reactor (2 mm) was tested by measuring the establishment of a steady state in two separate reactors working under the same operating conditions and with different preinocula. The steady-state concentration obtained was equal for the two reactors, and it was found to be stable over time (Supplementary Figure S4). With volume and thickness smaller than those used in this case, it was not possible to reach a steady state due to the reasons explained before. To the author’s knowledge, this is the first example of a continuously working photobioreactor for microalgal cultivation on such a small scale.

Accordingly, in these configurations, where a very small light path is present, it should be possible to discriminate the effect of light without the self-shading effect and mixing-induced light/dark cycle. From common knowledge, in reactors with high light paths, photoinhibition is visible from a certain critical value of light intensity. However, Figure 1 shows that photoinhibition occurs at lower values of light intensities as we move to shorter light paths. Moreover, the trend of the concentration vs. light curve changed with the light path; data at low light paths (from 2 to 8 mm) showed a steeper decrease in the biomass concentration for inhibition than those at high reactor depths (15 and 35 mm), suggesting that inhibition is heavily augmented in PBRs with short light path. For convenience, in Figure 1, 2–8 mm data are plotted on a 0–500 µmol m^−2^ s^−1^ *x*-axis while 15–35 mm on 0–1200 µmol m^−2^ s^−1^.

The decrease in reactor thickness from 35 to 15 mm turned into higher biomass concentrations at photosaturation. This behavior is well-confirmed by the literature (Richmond, 1996; Qiang et al., 1998; Zou and Richmond, 2000; Lee 2001; Richmond et al., 2003); at higher scales, a decrease in the light path corresponds to higher cell concentrations until a lower limit of 1–1.3 cm thickness. We found that this value represents a turning point since a further decrease in ultra-thin light paths (2–8 mm) showed a decrease in biomass concentration, confirming again a higher influence of inhibition than that expected at these scales. This is also reported by Zou and Richmond (2000), where higher inhibition was found in a thickness of 1 cm than in 3 cm. In the literature, there are no reports on biomass growth in continuous systems with light paths lower than 1–1.3 cm. For a batch cultivation system, Qiang et al. (1998) found an increase in biomass productivity from 200 to 7.5 mm; however, we experimentally verified that the behavior of batch and continuous systems at ultra-thin light paths is considerably different. Experimental results and previous considerations show that an indefinite decrease in the reactor thickness cannot lead to an increase in cultivating performances in continuous systems and suggest the existence of an optimal light path value (as conceptualized in other studies (Richmond et al., 2003; Zarmi et al., 2020).

### 3.2 Parameter estimation results and modeling

**FIGURE 2:**
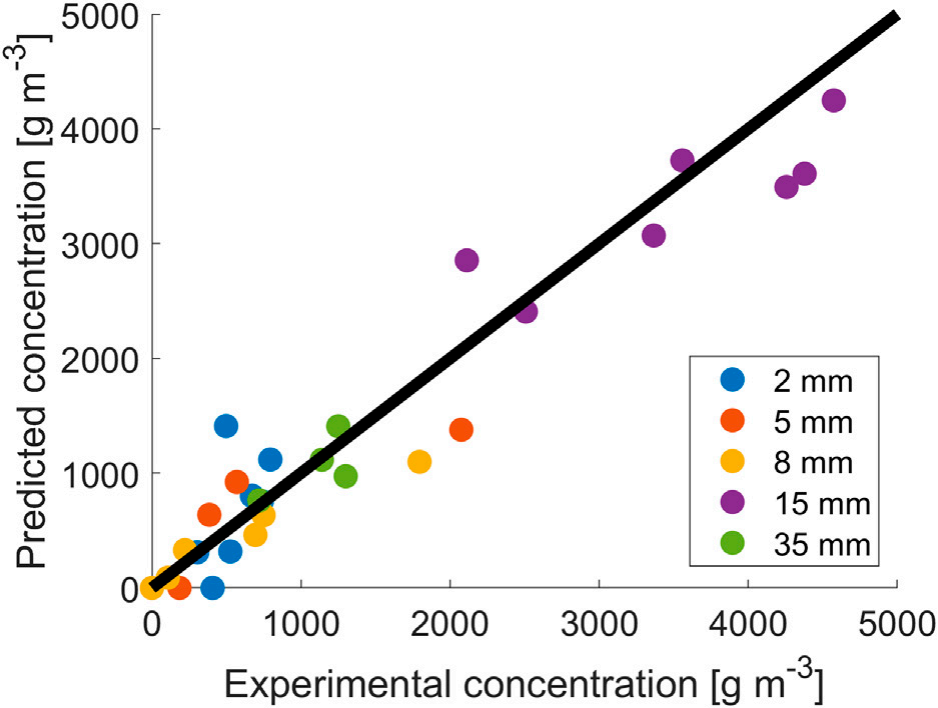
Fitting results for data at different reactor thicknesses.

To better describe the data obtained, a model was used to ascertain if the common growth modeling approach can be used at different light paths. As the model used was not able to reproduce, with a unique set of parameters, all the data at different thicknesses, the model parameters were retrieved separately for sets of data obtained with the same light paths. The values of $K_{I}$ and $k_{d}$ were found to be comparable for each series and similar to those reported by Barbera et al. (2020). Thus, the values of such parameters were fixed and $\mu_{max}$ , $I_{opt}$ , $\;k_{a}$ were estimated separately for each reactor thickness. A comparison between experimental and predicted values for each reactor is shown in Figure 2. Estimated parameter values are reported in Table 1. Of note, although $\mu_{max}$ estimates are quite stable (around 2 d^−1^), $I_{opt}$ and $\;k_{a}$ exhibit the largest variations among different culture depths.

**TABLE 1:** Fitted parameters for data at all reactor thicknesses.

mm	$\mu_{\max}$ [d^−1^]	$K_{I}$ [µmol m^−2^ s^−1^]	$I_{\mathrm{opt}}$ [µmol m^−2^ s^−1^]	$k_{d}$ [d^−1^]	$k_{a}$ [m^2^ g^−1^]
35	2.0	110	405	0.45	0.098
15	2.0	‘’	405	‘’	0.14
8	2.1	‘’	74	‘’	0.40
5	2.1	‘’	59	‘’	0.57
2	1.8	‘’	50	‘’	0.9

**FIGURE 3:**
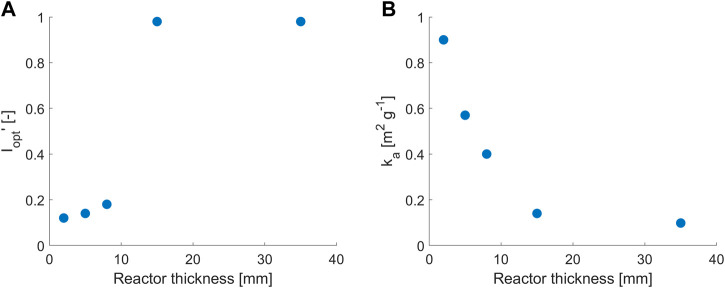
Trends of the non-dimensional value of **(A)** $I_{opt^{\prime }}$ and **(B)** $k_{a}$ for different reactor thicknesses. $I_{opt^{\prime }}$ is normalized on 413 µmol m^−2^ s^−1^ (Barbera et al., 2020).

Interestingly, the values of $I_{opt}$ and $\;k_{a}$ were found to be correlated to the reactor thickness, as shown in Figure 3. For convenience, in Figure 3A, $I_{opt^{\prime }}$ is shown, that is, $I_{opt}$ is normalized at the corresponding value of 413 µmol m^−2^ s^−1^ in Barbera et al. (2020). The increase of $I_{opt}$ for higher reactor thicknesses reflects qualitative experimental results in Figure 1: self-shading increases with culture depth, and the amount of light for optimal growth increases accordingly. The sharp increase in $k_{a}$ for lower reactor thickness needs additional considerations. Figure 3B shows that $k_{a}$ is constant for reactor thicknesses higher than 15 mm and reflects the experimental value of 0.09 m^2^ g^−1^ reported in Barbera et al. (2020), where cells experience mixing effects and move between different zones of the reactor at high and low irradiance values. For ultra-thin reactor light paths (2–8 mm), however, $k_{a}$ increases to account for higher light absorption from cells since they are not able to migrate to layers at a lower light intensity, where the excited photosystems may recover and reopen. The higher light absorption at small scales determines a greater inhibition than in thicker systems.

This behavior in ultra-thin PBRs is close to what is encountered in the outer layers of mass microalgal culture; cells in the outer layers are exposed to the highest irradiating conditions and subtract a considerable amount of light to the other layers (Kumar et al., 2021). In order to reduce this phenomenon, an extensive research effort has been performed to develop microalgae mutants with reduced light-harvesting apparatuses (Kumar et al., 2021). This also suggests that the standard Lambert–Beer law may not be able to represent the behavior at different light paths within a Haldane-like light model when the behavior of very thin photobioreactors needs to be described and that it may be inappropriate to scale down experimental results below some light path threshold.

## 4 Discussion

To understand the aforementioned limitations, we recall the two main adverse phenomena that hinder the net effect of light on microalgal growth: self-shading and mixing-induced light–dark cycles. In the model, self-shading is approached through light attenuation in the Lambert–Beer law (Eq. 1), while the effect of light–dark cycles is not considered. To obtain a measure of this phenomenon, Richmond et al. (2003) introduced the concept of cell travel time, that is, the average time required for cells to move back and forth in the reactor thickness, where they are exposed to different light conditions along the light attenuation profile. Richmond et al. (2003) defined some characteristic cell travel times, based either on random, diffusion-like motions or on back and forth movement through the optical path. The latter, which is called regular motion time, is more relevant in the context of this work and is defined as follows:

$$\;\tau_{t}=L/v_{cell},$$ (6)

where L (cm) is the optical path and $v_{cell}$ (cm s^−1^) is cell lateral velocity, assumed equal to the bubble velocity (30–50 cm s^−1^, in accordance with Richmond et al. (2003).

Cell travel time does not affect photosynthesis if it is one or two orders of magnitude higher than a characteristic PSU turnover time (about 10 ms for Richmond et al., 2003). Usually, this is not an issue. However, for very thin reactors, cell travel time starts to approach photosynthetic unit (PSU) turnover time, influencing light–cell interaction. Table 2 shows the cell travel times for the different reactor thicknesses used in our work. From 2 to 8 mm, travel times are very close to the PSU turnover time and are significantly smaller than travel times at 15 and 35 mm. This highlights that the behavior at 2–8 mm is different from that in 15–35 mm, and in the smaller scales, mixing-induced light–dark cycles could have a significant impact.

**TABLE 2:** Cell travel times for all reactor thicknesses.

	2 mm	5 mm	8 mm	15 mm	35 mm
Regular motion time [ms]	6.7	16.7	26.7	50	116.7

In an ideal situation for efficient light exploitation, cells should be exposed to high light conditions in the photic zone for a duration required for light reactions to occur and then move to the dark zone, being replaced by other cells from the dark zone ready to receive incoming photons (Richmond et al., 2003). However, if the time spent by cells in the dark zone is too low, dark reactions do not have enough time to occur and photosynthetic efficiency decreases (Zarmi et al., 2020). As introduced before, cells in ultra-thin reactors are exposed to strong illumination but, due to the flatter light profile and the absence of a proper dark zone, they do not fully recover from this strong light absorption, causing the photosynthetic efficiency to decrease. This is not the case in thick reactors, where cells are able to migrate to a dark zone and recover; this explains why photoinhibition in these systems is observed only under very high light conditions (or never, in some cases). Previous considerations suggest that not only light conditions cells are exposed to but also the duration of exposure of light along the attenuation light profile is necessary for the comprehension of cell–light interaction phenomena. To provide a systematic description, we can use the theory of the mathematical modeling of pulsed-light effects on microalgae: when cells move between zones with different irradiation, they are exposed to an effective illumination regime close to a pulsed light one (Zarmi et al., 2020). As in the study by Schulze et al. (2017), pulsed-light models have different inputs: intensity of the pulse, time of the light phase ( $t_{l}$ ), and time of the dark phase ( $t_{d}$ ). In an experimental pulsed-light apparatus, these inputs are usually set by a lamp, while in our case, we must calculate them with the aid of the travel time hypothesis in Richmond et al. (2003). The intensity of the pulse is here assumed equal to the light intensity of the light source. Here, we assume that the photic zone (expressed through the length coordinates $z_{ph}\;$ (m)) is the zone in the reactor in which light intensity is inhibiting, thus higher than $I_{opt}$ . Length $z_{ph}$ can be computed as

$$z_{ph}=-\frac{1}{k_{a}X_{out}}\log\frac{I_{opt}}{I_{0}}.$$ (7)

Light phase time for cell regular motion is calculated as

$$t_{l}=\frac{z_{ph}}{v_{cell}}.$$ (8)

Corresponding dark phase time is calculated as

$$t_{d}=\frac{W-z_{ph}}{v_{cell}},$$ (9)

where W is the the reactor thickness.

With light and dark phase times, the duty cycle ϕ (-) can be calculated as

$$\phi =\frac{t_{l}}{t_{l}+t_{d}}.$$ (10)

In our case, ϕ represents in proportion the time a single cell is exposed to inhibitory conditions. We observed that parameter *k*_*a*_ decreases until stabilizing at about *W* = 15 mm (Figure 3A), where its value is about 0.1 m^2^ g^−1^, which is consistent with other estimates in similar systems (Barbera et al., 2020). If that value is used to compute the duty cycle ϕ , it can be observed that $z_{ph}>W$ for 2–5–8 mm, and therefore ϕ can be assumed equal to 1 (Table 3). This means that in ultra-thin photobioreactors, cells are exposed to light continuously and cannot adequately recover from the high photon absorption in the high irradiance region. The consequent inhibition is artificially captured by the model by increasing $k_{a}$ , that is, by assuming less average light in the photobioreactor than there is actually.

**TABLE 3:** Duty cycle (non-dimensional) for all reactor thicknesses, computed for $k_{a}$ = 0.1 m^2^ g^−1^.

	2 mm	5 mm	8 mm	15 mm	35 mm
Duty cycle [-]	1	1	1	0.2	0.2

Previous considerations also show a touchpoint between thin-milli reactors and pulsed-light experiments toward the understanding and modeling of the effect of light on cell growth and confirm the importance of considering light–dark mixing cycles to ensure the scalability of laboratory results to higher-scale systems. Indeed, Eqs. 7–10 may represent a starting point to integrate the effect of mixing cycles in traditional light modeling in microalgal growth.

## 5 Conclusion

In high-scale microalgal cultivation systems, self-shading and mixing-induced light–dark cycles heavily hinder the true effect of light interaction with cells. To minimize these phenomena, we designed, built, and operated ultra-thin continuous photobioreactors. Experiments showed that at very low light paths (2–8 mm), high inhibition occurred at low irradiance values. We used experimental data from different reactor thicknesses to retrieve parameters of a Haldane-like model, and the trend of the fitted Lambert–Beer constant shows that there could be issues in the capability of the model to scale up experimental results at ultra-thin light paths. We infer that this is due to the averaged nature of the model that does not consider the effect of mixing-induced light–dark cycles. Indeed, the computation of parameters from the theory of mathematical modeling of pulsed-light effects on microalgae suggested that only in ultra-thin light paths (2–8 mm), the “real” continuous light regimen occurs, while in thicker light paths (15–35 mm) cells are subjected to an effective pulsed light one due to mixing-induced light–dark cycles.

## Data Availability

The raw data supporting the conclusion of this article will be made available by the authors, upon request. Requests to access these datasets should be directed to Eleonora Sforza, eleonora.sforza@unipd.it.

## Nomenclature

I: Light intensity, µmol m^−2^ s^−1^
$I_{0}$: Incident light intensity, µmol m^−2^ s^−1^
$I_{opt}$: Optimal light intensity, µmol m^−2^ s^−1^
$I_{opt^{\prime }}$: Normalized optimal light intensity, −
$k_{a}$: Biomass light absorption coefficient, m^2^ g^−1^
$k_{d}$: Specific decay rate, d^−1^
$K_{I}$: Light half-saturation constant, µmol m^−2^ s^−1^
L: Optical path length, cm
$r_{x}$: Biomass production rate, g m^−3^ d^−1^
${\bar{r}}_{x}$: Average biomass production rate, g m^−3^ d^−1^
$t_{l}\;$: Light phase time, ms
$t_{d}$: Dark phase time, ms
$v_{cell}$: Cell lateral velocity, cm s^−1^
$V_{R}$: Volume of the reactor, m^3^
$\dot{V}$: Volumetric liquid flowrate, m^3^ d^−1^
W: Reactor thickness, m
$X_{out}$: Biomass concentration, g m^−3^
z: Axial coordinate of the reactor depth, m
$z_{ph}$: Length of the photic zone, m

### Greek letters

$\mu_{max}$: Maximum biomass-specific growth rate, d^−1^
τ: Residence time, d
$\tau_{t}$: Cell travel time, ms
ϕ: Duty cycle, −

### Abbreviations

CSTR: Continuous stirred tank reactor
PBR: Photobioreactor
PSU: Photosynthetic unit
TLC: Thin-layer cascade
