# Supplementary material for: Microalgae growth in ultra-thin steady-state continuous photobioreactors: assessing self-shading effects
Source: Front Bioeng Biotechnol. 2022 Aug 11;10:977429. doi: 10.3389/fbioe.2022.977429 (PMC9402969; doi:10.3389/fbioe.2022.977429)
Supplement: Supplementary file 1 [file DataSheet1.docx]

Supplementary Material

# Photobioreactors

Figures S1 and S2 respectively show the 2 mm and 5 mm light path photobioreactors employed in this work. Notice that the shape of 5 and 8 mm thickness PBRs are similar. Figure S3 shows a scheme of our cultivation system.


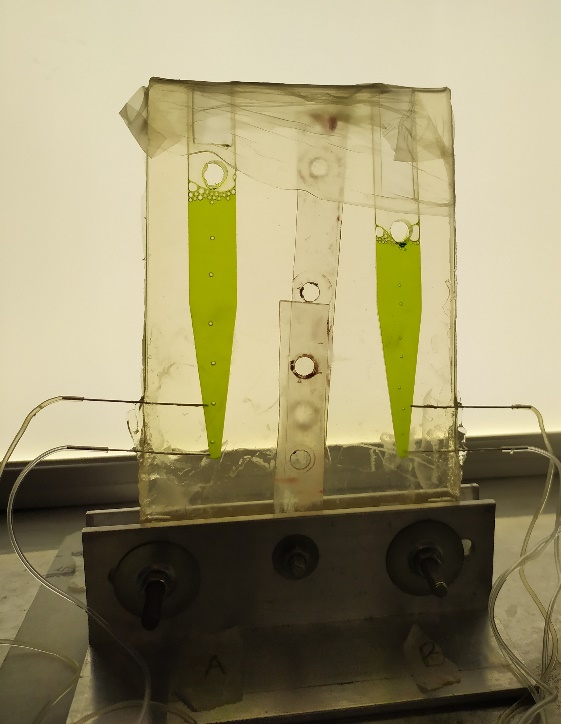


Supplementary Figure 1. 2 mm light path photobioreactor.


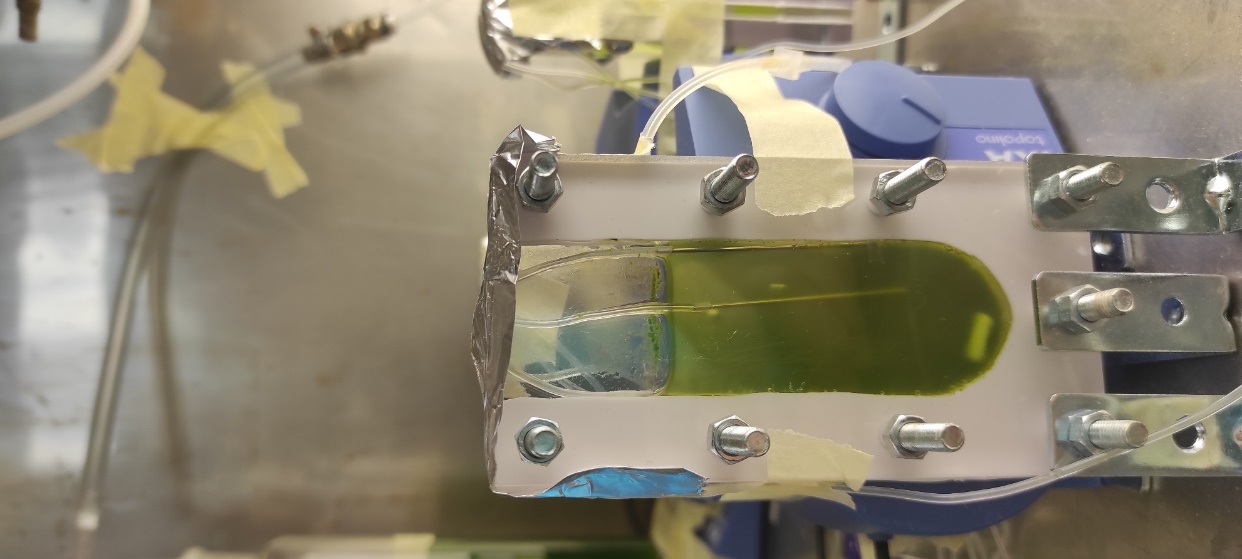


Supplementary Figure 2. 5 mm light path photobioreactor.


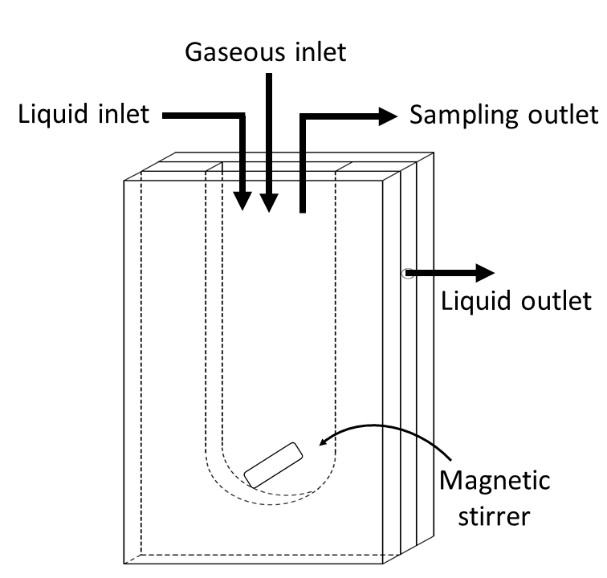


Supplementary Figure 3. A schematic representation of our cultivation system

# Medium composition

Table S1 reports the composition of the BG11 2x employed in this work.

**Supplementary Table 1.** Growth medium composition of BG11 2x.

| **Component** | **Concentration [mg L^-1^]** |
| --- | --- |
| $\boldsymbol{N}\boldsymbol{a}_{\mathbf{2}}\boldsymbol{Mg} \boldsymbol{EDTA}$ | 2 |
| $\boldsymbol{Ferric} \boldsymbol{Ammonium} \boldsymbol{Citrate}$ | 12 |
| $\boldsymbol{Citric} \boldsymbol{Acid}\boldsymbol{\cdot}\boldsymbol{H}_{\mathbf{2}}\boldsymbol{O}$ | 12 |
| $\boldsymbol{CaC}\boldsymbol{l}_{\mathbf{2}}\boldsymbol{\cdot}{\mathbf{2}\boldsymbol{H}}_{\mathbf{2}}\boldsymbol{O}$ | 72 |
| $\boldsymbol{MgS}\boldsymbol{O}_{\mathbf{4}}\boldsymbol{\cdot}{\mathbf{7}\boldsymbol{H}}_{\mathbf{2}}\boldsymbol{O}$ | 150 |
| $\boldsymbol{K}_{\mathbf{2}}\boldsymbol{HP}\boldsymbol{O}_{\mathbf{4}}$ | 61 |
| $\boldsymbol{H}_{\mathbf{3}}\boldsymbol{B}\boldsymbol{O}_{\mathbf{3}}$ | 5.72 |
| $\boldsymbol{MnC}\boldsymbol{l}_{\mathbf{2}}\boldsymbol{\cdot}{\mathbf{4}\boldsymbol{H}}_{\mathbf{2}}\boldsymbol{O}$ | 3.62 |
| $\boldsymbol{ZnS}\boldsymbol{O}_{\mathbf{4}}\boldsymbol{\cdot}{\mathbf{7}\boldsymbol{H}}_{\mathbf{2}}\boldsymbol{O}$ | 0.44 |
| $\boldsymbol{CuS}\boldsymbol{O}_{\mathbf{4}}\boldsymbol{\cdot}{\mathbf{5}\boldsymbol{H}}_{\mathbf{2}}\boldsymbol{O}$ | 0.16 |
| $\boldsymbol{COCl}\boldsymbol{\cdot}{\mathbf{6}\boldsymbol{H}}_{\mathbf{2}}\boldsymbol{O}$ | 0.1 |
| $\boldsymbol{N}\boldsymbol{a}_{\mathbf{2}}\boldsymbol{Mo}\boldsymbol{O}_{\mathbf{4}}\boldsymbol{\cdot}{\mathbf{2}\boldsymbol{H}}_{\mathbf{2}}\boldsymbol{O}$ | 0.78 |
| $\boldsymbol{N}\boldsymbol{a}_{\mathbf{2}}\boldsymbol{C}\boldsymbol{O}_{\mathbf{3}}$ | 40 |
| $\boldsymbol{NaN}\boldsymbol{O}_{\mathbf{3}}$ | 3000 |

# Reproducibility of steady states at 2 mm

To our knowledge, the cultivation of microalgae in a continuous PBR with 2 mm light path represents a novelty of this work. For all the experiments (and especially those at 2 mm thickness), we ensured the stability of the steady state by maintaining a steady for at least three times the residence time. Moreover, we performed two replicas of each experiment at 2 mm with the same input conditions, at different times and from different preoinocula. An example of the results is reported in Figure S4.


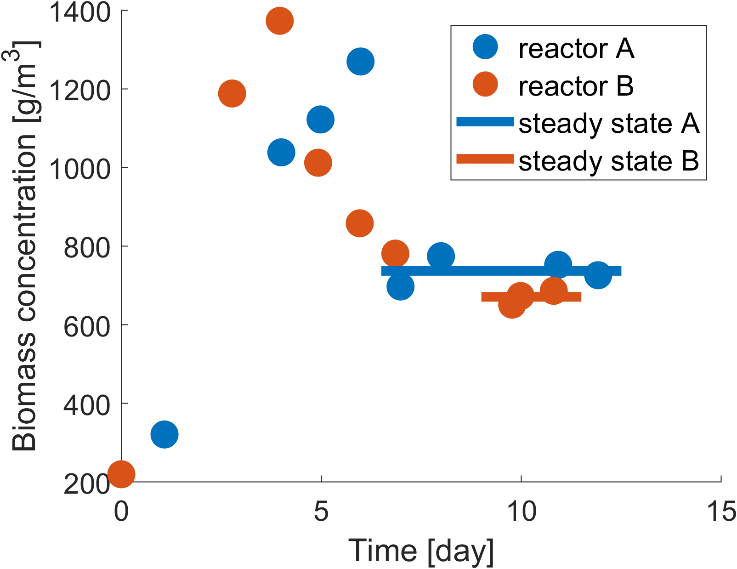


Supplementary Figure 4. Experimental cell growth data for two replicas (A and B) at 50 µmol m^-2^ s^-1^ and 1.5 d residence time with steady state values.
